# Predicting humor effectiveness of robots for human line cutting

**DOI:** 10.3389/frobt.2024.1407095

**Published:** 2024-10-29

**Authors:** Yuto Ushijima, Satoru Satake, Takayuki Kanda

**Affiliations:** ^1^ Human Robot Interaction Laboratory, Department of Social Informatics, Kyoto University, Kyoto, Japan; ^2^ Deep Interaction Laboratory, Advanced Telecommunications Research Institute International, Kyoto, Japan

**Keywords:** human-robot interaction, humor, low moral behaviors, crowdsourcing, machine learning

## Abstract

It is extremely challenging for security guard robots to independently stop human line-cutting behavior. We propose addressing this issue by using humorous phrases. First, we created a dataset and built a humor effectiveness predictor. Using a simulator, we replicated 13,000 situations of line-cutting behavior and collected 500 humorous phrases through crowdsourcing. Combining these simulators and phrases, we evaluated each phrase’s effectiveness in different situations through crowdsourcing. Using machine learning with this dataset, we constructed a humor effectiveness predictor. In the process of preparing this machine learning, we discovered that considering the situation and the discomfort caused by the phrase is crucial for predicting the effectiveness of humor. Next, we constructed a system to select the best humorous phrase for the line-cutting behavior using this predictor. We then conducted a video experiment in which we compared the humorous phrases selected using this proposed system with typical non-humorous phrases. The results revealed that humorous phrases selected by the proposed system were more effective in discouraging line-cutting behavior than typical non-humorous phrases.

## 1 Introduction

**FIGURE 1:**
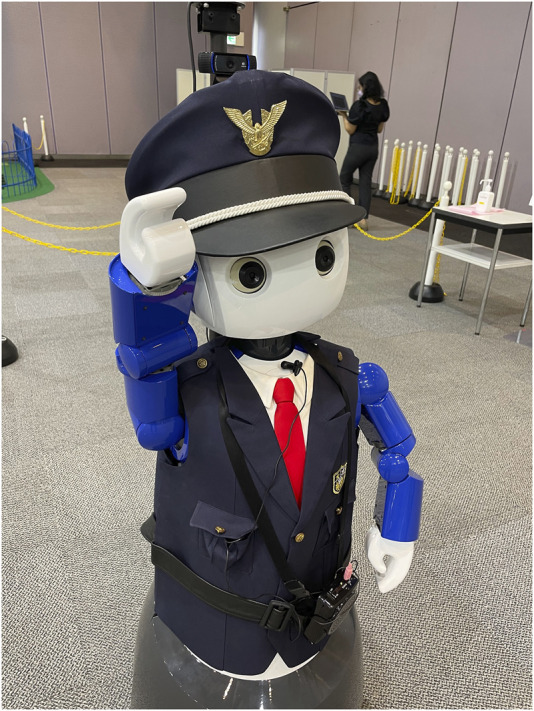
Security guard robot.

In recent years, there has been remarkable progress in artificial intelligence and robotics technology, leading to an increase in robots working in place of humans. One such example that is anticipated to play a significant role in the future is the security robot depicted in Figure 1. Tasks expected to be performed by security robots include forming and guiding queues at events, and detecting and addressing low moral behaviors such as littering or smoking in non-designated areas, similar to the tasks performed by human security guards.

Security guard robots must be able to persuade people who engage in low moral behavior to refrain from such activities. In this study, we focus on addressing line cutting as a low moral behavior that security robots must address. Line cutting is a common, low moral behavior that occurs at many events and venues, both indoors and outdoors.

However, getting people to obey a robot’s requests is challenging, especially when they are engaged in low moral behavior. However, getting people to obey a robot’s requests is challenging, especially when they are engaged in low moral behavior. In the study conducted by Schneider et al., when a robot called on people to stop using their smartphones while walking, about half of the 160 participants did not comply with the robot’s request (Schneider et al., 2022). Additionally, children who disrupt a robot’s work for amusement often continue their behavior despite the robot’s requests to stop (Brščić et al., 2015). There is a limited amount of research that specifically addresses effective methods for dealing with low moral behaviors. We need to find an effective method using dialogue for such issues. Research that specifically addresses effective methods for dealing with low moral behaviors is limited. We need to find an effective method for such issues through dialogue.

As a creative approach to robot persuasion, this study suggests that robots can use humor to effectuate behavior change. Humans often employ humor to persuade others or resolve issues. For example, the following is a humorous tactic: “Hey there. I see you’re in a rush. I hear that the back of the line is actually a very nice place to relax!” Humor has long been explained through various theories (Shurcliff, 1968; Forabosco, 1992), with a common thread among them being the use of techniques different from ordinary sentences. Humor provide surprise or insight to the audience, and can lead to stress reduction, strengthened group cohesion (Mesmer-Magnus et al., 2012), capturing the audience’s attention, and enhancing the trust and likability of the speaker (Weinberger and Gulas, 1992). Given the positive effects of humor, we propose incorporating humor into robot persuasion strategies.

However, effectively using humor is challenging for robots. When a robot considers delivering humorous persuasion, it must predict the effectiveness of that humor. While previous research has revealed methods for recognizing whether a statement is humorous (Yang et al., 2015; Zhang and Liu, 2014; Chen and Soo, 2018), predicting its effectiveness remains unexplored territory. Furthermore, as evident in human interactions, predicting effectiveness is a challenging task, even for humans.

Therefore, this study aims to predict when using humor would be effective for robots in addressing line-cutting behavior. In this study, we adopt machine learning to predict the effectiveness of humor. In using machine learning, we hypothesize the importance of applying situational context and discomfort as parameters. Additionally, we constructed a system to select the best humor for line-cutting behavior using this predictor and demonstrate through video experiments that humor selected in this way is superior in effectiveness to the typically used non-humorous persuasion.

## 2 Related works

### 2.1 Using humor for persuasion in human-human interaction

Firstly, regarding persuasion using humor, Walter et al. conducted a meta-analysis of 89 studies across the various fields in which humor has been researched, and found that humor has a significant effect on persuasiveness (Walter et al., 2018). Many of the reserches that discuss the effect of humor on persuasion focus on advertising (Weinberger and Campbell, 1991; Duncan and Nelson, 1985; Zhang and Zinkhan, 2006). Particularly, in fear advertisements promoting smoking cessation or safe driving, the use of humor, such as cartoons, enhanced the effect (Mukherjee and Dubé, 2012). This is evident in fear appeal ads where the use of humor mitigates defensive reactions, even when fear appeals alone might lead to decreased effectiveness by triggering defensive responses in the audience.

Additionally, the use of humor in politics and its political influence has also been highlighted (Meyer, 2000). Hakoköngäs et al. argued that humor are used as Internet meme to crystallize their arguments in an easily shareable and concise form, which makes the memes useful tools in persuasion and mobilization, as well as attracting new audiences (Hakoköngäs et al., 2020). Humor is also used as a tool for moral criticism (Dadlez, 2011), and satire is one of the oldest forms of humor (LeBoeuf, 2007). Additionally, humor is often seen as a fundamental characteristic of irony (Garmendia, 2014). Moreover, humor is known to yield various benefits in education (Wallinger, 1997). Lyttle has practically demonstrated the benefits of humor, including cartoons and irony, in business ethics training (Lyttle, 2001).

### 2.2 Persuasion by robots for low moral behavior

As social agents, robots must be able to persuade humans, perhaps in a similar manner to how humans do it themselves. Human-robot interaction (HRI) studies often discuss how robots can effectively persuade humans. Siegel et al. concluded that men are more likely to comply with such requests from woman-designed robots (Siegel et al., 2009). Chidambaram et al. demonstrated the effectiveness of bodily cues, such as proximity, gaze, and gestures (Chidambaram et al., 2012). Ham et al. focused on gaze and gestures, revealing that using the former increases the persuasiveness of a robot’s spoken content (Ham et al., 2015). Winkle et al. concluded that, when a robot demonstrates goodwill and similarity to the user, it enhances its persuasiveness (Winkle et al., 2019).

However, resolving low moral behavior that occurs during a robot’s tasks is still challenging. For example, Brščić et al. pointed out the occurrence of security guard robot abuse by children and demonstrated that this could be avoided by keeping children away from the robot (Brščić et al., 2015). In this research, the robot could not overcome the abuse through interactions and it was very difficult for the robot to persuade children not to abuse it. Mizumaru et al. also revealed that, when a robot admonishes people for walking while using their smartphones, many people ignore the robot (Mizumaru et al., 2019). Schneider et al. revealed that such people use trivialization as the reason for ignoring the robot (Schneider et al., 2022). Additionally, Agrawal and Williams revealed that the degree of authority people perceive in robots has little influence on whether they obey the robots or not (Agrawal and Williams, 2017).

Furthermore, robots are susceptible to aggressive behavior based on their design. Keijsers and Bartneck examined the influence of dehumanization primes and found that when participants attributed fewer mental attributes to the robot, they tended to exhibit more aggressive responses towards it (Keijsers and Bartneck, 2018).

### 2.3 Using humor in human-robot interaction

As an example of robots’ tasks using humor skills, some robots that perform stand-up comedy (Katevas et al., 2015) or Japanese Manzai (Hayashi et al., 2008). These robots construct scenarios based on the performances of human professionals. Katevas et al. revealed the relationship between the effectiveness of punchline, gaze and gesture and happiness of the audiences (Katevas et al., 2015). Hayashi et al. revealed that robots could get higher evaluations on presence and overall impression than amateurs (Hayashi et al., 2008).

The usefulness of humor in service robots has also been demonstrated. Yang et al. have shown that when a robot fails to deliver a service and the failure is not severe, using humor can result in higher evaluations compared to standard responses (Yang et al., 2022). Additionally, Green and colleagues have demonstrated that when a robot uses humor in the event of a task failure, it can minimize the negative impact of the failure on perceived warmth, competency, and the robot’s status as a teammate (Green et al., 2022).

Another example is the chatbots developed by Augello et al. (2008), which recognize humor and respond appropriately to user comments to increase chatting quality. These are based on three pre-defined classifications of humor, such as alliteration, antinomy, and adult slang, using the open source chatbot A.L.I.C.E. and special artificial intelligence markup language (AIML) categories.

### 2.4 Humor recognition

In previous research, computational approaches to humor have primarily focused on humor expressed through text and the main computational approaches use machine learning. Yang et al. revealed the combination of word2vec and highest common factor (HCF) to be the best learning method compared to several recognition approaches, such as bag-of-words, word2vec, the language model, and a combination of four latent structures (incongruity, ambiguity, interpersonal effect, and phonetic style) and k-nearest neighbors (KNN) features such as human centric features (Yang et al., 2015). They also proposed a method to automatically extract anchors that enable humor in a sentence.

Zhang and Liu concluded that lexico-semantic and morpho-syntactic features are the most useful for distinguishing between humorous and non-humorous tweets (Zhang and Liu, 2014). Chen and Soo also constructed and collected four datasets with distinct types of jokes in both English and Chinese, and conducted learning experiments on humor recognition using a convolutional neural network (CNN) (Chen and Soo, 2018).

In using humor effectively, it is not enough to merely recognize humor; predicting its effects is also crucial, just like with humans. Previous works have shown successful use of machine learning for humor recognition, but further investigation is needed to determine how to estimate the effects of humorous phrases, a topic that has been rarely addressed.

Therefore, in this study, we developed a machine learning framework to predict the effectiveness of humorous phrases for low moral behavior.

## 3 Construction of humor dataset

**FIGURE 2:**
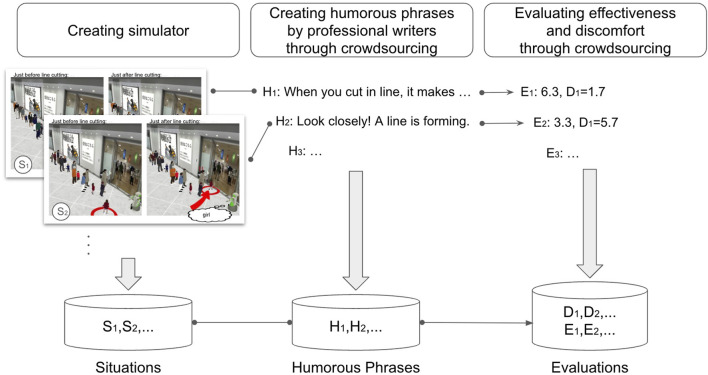
Overview of the dataset construction approach.

To predict the effectiveness of humor for addressing line cutting, we need to construct a dataset regarding such effects. We consider that, for humor to be effective, it must be situationally appropriate and not cause too much discomfort to the line-cutting offenders. We constructed our dataset with this in mind. Figure 2 outlines the process of our dataset construction. We used situations data for the machine learning input for situational appropriateness, and learned and predicted discomfort as the machine learning input for avoiding discomfort.

Given the difficulty of directly observing interactions between robots and humans regarding line cutting, we employed simulation and crowdsourcing. The simulator generated a large number of line-cutting scenarios, which were then combined with the humorous phrase data collected through crowdsourcing. We further used crowdsourcing to evaluate the discomfort and effectiveness associated with this combination.

### 3.1 Consideration of important factors for humor effectiveness prediction

To construct a dataset for machine learning that predicts the effectiveness of humor, we need to consider several crucial elements before proceeding.

First, the effectiveness of humor heavily depends on the context in which it is delivered. For example, phrases that work well with children may not necessarily be effective with adults. “If you cut in line, your parents might be taken away by the police” is effective with children but may not resonate with adults. Similarly, phrases effective between couples may not apply to others, like “Compared to the time you have spent together, the waiting time is trivial.”

Second, it is crucial that the humor not cause discomfort. Even if humor is contextually appropriate, its delivery may offend the recipient. For example, phrases like “Someone needs to repeat their moral education! “or “You may look like an adult, but your actions are quite childish” might anger the recipient and fail to persuade. Therefore, such phrases should be avoided.

Hence, in constructing the dataset, it is essential to evaluate various phrases in different contexts, considering both their effectiveness and the potential discomfort they may cause.

### 3.2 Data representation

We created a dataset (Table 1) that consists of combinations of text (humorous phrases), a list of parameters representing the situations, and discomfort and effectiveness values. For the text $H_{i}$ , multiple situations, $S_{j}$ , $S_{j+1}$ , …, are associated. For each text-situation pair, discomfort and effectiveness values ( $D_{j}$ , $U_{j}$ ) are provided. Situations are represented by 72-dimensional vectors (Section 4) that describe the attributes of the people in line and those who are cutting it.

**TABLE 1:** Data representation.

Humorous phrases	Situation vector	Discomfort value	Effectiveness value
$H_{i}$	$S_{j}=\left[s_{j,1},\ldots ,s_{j,72}\right]$ $S_{j+1}=\left[s_{j+1,1},\ldots ,s_{j+1,72}\right]$ ⋮	$D_{j}$ $D_{j+1}$ ⋮	$E_{j}$ $E_{j+1}$ ⋮
⋮	⋮	⋮	⋮

### 3.3 Creating a simulator to reproduce various line-cutting behaviors

Collecting data on instances of line cutting requires much more time compared to gathering data on regular interactions. This is because the occurrence frequency of line-cutting behaviors, classified as low moral behaviors, is lower than that of normal behaviors. In our preliminary observations, we confirmed a low incidence rate. We used a security robot to guide a line at an event, but the number of line-cutting behavior is during this task remained below one per day. Due to the low frequency of occurrence, it becomes exceedingly challenging to test various phrases in different situations, and conduct observations and interviews regarding their discomfort and effectiveness in real environments.

**FIGURE 3:**
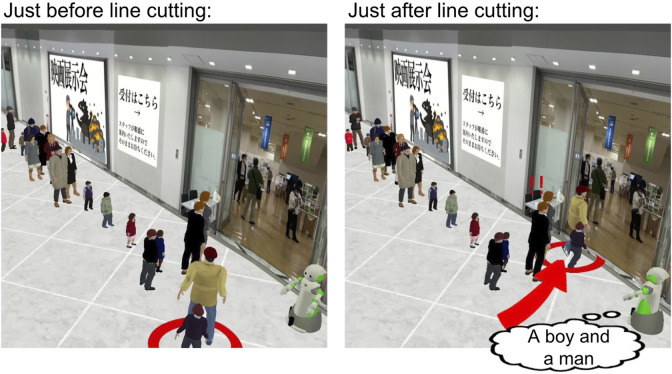
An example of a simulated scene of line cutting.

For this reason, we constructed a simulator (Figure 3) as an alternative to collecting data in the real world. This simulator replicates the scenario based on interviews with three security guard experts, where 11 groups of customers are lining up at a fictional movie exhibition, along with the behavior of one customer or a pair of customers cutting in line at the front.

We developed the simulator based on a publicly available simulator Blender-based software (Echeverria et al., 2011). We developed 33 different 3D avatars with varying appearances in terms of age, clothing, and gender, a 3D model of a real commercial facility and the robot’s 3D model adopted to match that of an actual security guard robot.

In the simulator, firstly, regular avatars execute action modules to join the line. After all the regular avatars have lined up, low moral avatar appears and cuts in line at the front. The appearance of these avatars is randomly assigned. When denoting the position of a customer group in the line as n (first avatars are n=0 and line-cutting avatars are the last number n=11 ), each group of these avatars has the following parameters:

• Member Alone or in a pair. If the customer is alone, $s_{6*n+4}-s_{6*n+6}$ is [0,0,0].

• Gender Man or woman, expressed by $s_{6*n+1}$ , $s_{6*n+4}$ .

• Age Child, adult, or older adult expressed by $s_{6*n+2}$ , $s_{6*n+5}$ .

• Clothing Formal or casual, expressed by $s_{6*n+3}$ , $s_{6*n+6}$ .

For example, in the scenario depicted in Figure 3, the front of the line consists of two adult men wearing suits, denoted as $s_{1}-s_{6}$ , with the attributes Man, Adult, Formal, Woman, Adult, Formal. There is also a girl standing third in line, denoted as $s_{13}-s_{18}$ , with the attributes Woman, Child, Casual, 0, 0, 0. By varying these parameters, it is possible to generate numerous simulated images. This information is recorded as a situation vector for later use in machine learning (Section 4).

We highlighted which customer is engaging in line cutting for clarity. Additionally, a red arrow is used to indicate the line cutting for emphasis. When line cutting occurs, two exclamation marks “!!” is displayed above the head of the first customer group. This is intended to prevent misunderstandings when observing the simulator, ensuring it is understood that the action is unintentional for the first customer group.

The robot stationed at the entrance of the exhibition hall tracks the customer with its eyes when a line-cutting incident occurs. Due to the limitations of the simulator specifications, where it may be difficult to discern the gender or age of avatars, the attributes of the line-cutting avatar are explicitly stated within a speech bubble displayed by the robot.

### 3.4 Creating humorous phrases through crowdsourcing

We recruited 100 professional writers for high quality humorous phrases through crowdsourcing. Generating humorous phrases requires significant creative ability, so we chose to use writers rather than security personnel or the general public. This crowdsourcing was conducted by a Japanese company “Komagane Telework Office Koto” and all writers were Japanese speakers. In this task, each writer received a uniform payment of 5,000 JPY. The data collected are anonymized and all rights to the data obtained through this crowdsourcing belong to us.

We conducted the data collection in three steps: the writers received an explanation of the task, generated humorous phrases, and generated typical phrases.

First, we explained the task of proposing humorous phrases. We presented the simulator created in Section 3.3 as an example and described it as a scenario where a line is forming at an exhibition. We explained that when a line-cutting incident occurs, it happens at the front of the line and, if the attributes of the avatars are unclear, writers can refer to the speech bubble displayed by the robot. Additionally, we provided examples of humorous phrases that the writers should propose, as well as examples of completely non-humorous phrases that we would reject.

Next, we provided the writers with five images of low moral behavior in the simulator, which are identical to those depicted in Figure 3. For each behavior, they wrote at least one humorous phrase related to the situation, ensuring a total of 15 or more phrases. Even for professionals, proposing humorous phrases requires a significant amount of time and, considering the cost of crowdsourcing, we set minimum conditions for the total number. Additionally, to collect lines for various situations, we established minimum conditions for each specific situation.

Finally, we asked them to write three typical phrases that a human security guard would be likely to say, such as “Please do not cut in line.” These typical phrases were collected for evaluating the effectiveness of the humorous phrases selected using the humor effect predictor in Section 7.

Regarding the quality of the phrases, we only considered responses that raised contractual issues on the crowdsourcing platform, meaning those that were deemed not to have been completed properly. Such responses included those that were very similar to the examples we provided, contained multiple nearly identical phrases within the suggestions, or consisted of non-humorous phrases typically used by security personnel. When this problem happen, we did not provide rewards to them, and their evaluations was shared with the platform. This is because it is desirable to have some variation in humor quality within the dataset for machine learning purposes and these variations in quality would be evaluated later when evaluating for discomfort and effectiveness, which would not affect the machine learning process.

### 3.5 Evaluating Humor’s effectiveness and discomfort

We combined various situations generated by the simulator created in Section 3.3 with the humorous phrases collected in Section 3.4, and evaluated the discomfort and effectiveness of each phrase for each situation. A total of 13,000 situations were generated, with 500 phrases assigned to each situation equally. This allocation was done randomly.

For each combination, discomfort and effectiveness data were evaluated through crowdsourcing, which was conducted by the same Japanese company as in Section 3.4, with all respondents being Japanese speakers. All respondents were compensated 600 JPY. The data collected are anonymized and do not identify individuals, and all rights to the data belong to us.

First, the respondents were told that the simulator replicated an exhibition and its queue where instances of cutting in line occurred despite instructions from the security guard robot. Furthermore, the robot that noticed the line cutting will attempt to discourage this behavior. Additionally, respondents were instructed to respond from the perspective of a customer cutting in line in the situations replicated by the simulator.

Next, the respondents reviewed 35 types of phrases along with simulated combinations and provided responses to the following evaluating questions for the robot’s phrases using a 7-point Likert scale:

• Effectiveness: “Would you consider stopping the low moral behavior if you heard this phrase?”

• Discomfort: “How uncomfortable does this phrase make you feel?”

Considering the potentially large individual difference in humor perception, each combination was assigned to three annotators and the average values of the discomfort and the effectiveness were used as the final values.

In addition, we established two criteria to ensure the quality of the responses. First, we provided two combinations of phrases and situations common to all participants, asking them whether they felt these phrases were humorous. These two phrases were entirely non-humorous businessy phrases typically used by security guards. Participants who responded that these phrases were humorous were rejected. Next, the participants were asked to provide the ages and genders of the people cutting in line for each phrase and simulator combination, and participants who did not answer correctly were rejected.

### 3.6 Result

We collected a total of 500 humorous phrases and 300 typical phrases. Each humorous phrase was associated with 26 situations with associated discomfort and effectiveness values. In total, there were 13,000 data instances; 500 phrases each with 26 situational combinations, and discomfort and effectiveness values for each combination included in our dataset. Examples are shown in Tables 2, 3.

**TABLE 2:** Representative examples of high-effectiveness humor.

Humorous phrases	Discomfort	Effectiveness
When you cut in line, it makes the artwork sad and it ends up crying So, please wait in line to keep the artwork smiling	1.00	7.00
Look closely! A line is forming	1.33	7.00
Hey there, dads! How about getting in line and aiming to be handsome dads?	2.67	6.67

**TABLE 3:** Representative examples of low-effectiveness humor.

Humorous phrases	Discomfort	Effectiveness
Sorry, these customers are not sorted by how soon they will die	6.67	2.33
Let’s receive moral education again!	7.00	1.67
Could you please share your thoughts on the act of cutting in line?	6.33	1.33

A total of 2,122 respondents participated in the evaluating process, with 26.5% of the responses being rejected. This was due to respondents inaccurately answering the validation questions they were presented with. In other words, these respondents either evaluated typical non-humorous phrases as humorous or inaccurately identified the attributes of the line-cutting avatars displayed in the simulator.

## 4 Learning effectiveness of humorous phrases

### 4.1 Overview

**FIGURE 4:**
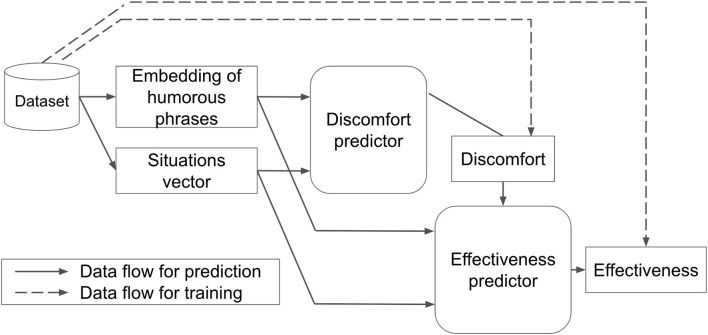
Learning overview.

Figure 4 illustrates our machine learning architecture. It takes a humorous phrase and situation as inputs and then predicts the effectiveness of the phrase for stopping line-cutting behavior in the given situation. The key idea of this architecture is the inclusion of a discomfort predictor. It predicts how much discomfort the given phrase may cause in the given situation, which is then used for the input for the effectiveness predictor. Both predictors are trained using the humor dataset described in Section 3.

### 4.2 Embedding humorous phrases

The humorous phrases were converted into feature vectors using embedding models provided by OpenAI: “text-embedding-ada-002” (OpenAI, 2023). This allowed us to incorporate the similarity between phrases into vector representations, making it possible to use this information for learning as well. We used 1536-dimensional vector representations of each humorous phrase as a feature set.

### 4.3 Situation vectors

In this study, we treat the situation vector as an input to the neural network. This is because it is difficult to make situational prediction using simple if/else statements or SVM. Regarding the situation vector, there are 90 possible combinations for each set of avatars in line and avatars cutting in it. It is necessary to use machine learning to handle these combinations.

Then, we represented the situations as vectors and then performed feature engineering on them to make the vectors sufficiently short. That is, we started the situation vectors from the attributes list of people lining up and cutting in line in the simulator, as shown in Section 3.3. However, this initial representation of the situation vectors contained many elements that could introduce noise into the learning process.

To mitigate this, we reduced dimensions through feature engineering. For instance, the data about the gender of the 10th person in the line of the situation vector was unlikely to be highly relevant to the selection of humor. We conducted our reduction in dimension as follows: First, the situation vector, which initially included information about everyone in the line, not just the line-cutter, was transformed into 18 features. Second, using these 18 features, we performed feature engineering to derive the following six features that most positively impacted the machine learning: whether one person or two cut the line; whether children were included; whether they were considered family; whether they were a parent and child pair; whether the parent with the child was the mother; and whether other children were in the line.

### 4.4 Discomfort predictor

We constructed the discomfort predictor to take the features of the phrases and the situation vectors as the input and predict discomfort values as the output. We selected a neural network from various machine learning methods because neural networks learn well from high-dimensional data. The input layer consisted of 1,542 dimensions, which included the 1536-dimensional phrase features of the dialogue and the 6-dimensional situation vectors improved through feature engineering. The hidden layers consisted of four layers with 768-512-256-128 nodes. The output was a 1-dimensional discomfort score that takes continuous values from 1 to 7.

### 4.5 Effectiveness predictor

The effectiveness predictor is a similar neural network to the discomfort predictor with the exception of taking one additional input of discomfort value. That is, the effectiveness predictor is constructed to take the features of the phrases, the situation vectors, and the predicted discomfort value as the input and predicts effectiveness values as the output. The input layer consisted of 1,543 dimensions, which included the 1536-dimensional phrase features of the dialogue, the 6-dimensional situation vectors improved through feature engineering, and the 1-dimensional discomfort value. The hidden layers consisted of four layers with 768-512-256-128 nodes. The output had a 1-dimensional effectiveness score that takes continuous values from 1 to 7.

## 5 Evaluation

We evaluated our proposed humor learning approach, which incorporates the prediction of the situation and discomfort along with the phrase as input. In this machine learning process, discomfort is predicted using the phrase and situation as inputs, and then this discomfort, along with the phrase and situation, is used as input to predict the final effectiveness. To assess its effectiveness, we compared it with three methods: a baseline approach, learning only the phrases, and learning both phrases and situations. We verified that there were significant differences among all these methods. Additionally, we highlighted notable examples where learning from the situation and discomfort predictions was particularly effective in the proposed method.

### 5.1 Methods

We developed the proposed method based on the considerations in Section 3.1, which specifically focus on the use of the situation and the expected discomfort in addition to the phrase itself as the input for predicting the effectiveness of the humorous phrases. Our aim is to validate our idea in the evaluation. Therefore, we compared the proposed method with methods that consider only the phrases and not the situation and discomfort. We compared the following three methods with our proposed method based on the mean squared error (MSE) between the predicted effectiveness values and the ground truth values.

• Proposed Method (Phrases + Situations + Discomfort): We used the proposed learning method described in Section 4.

• Phrases + Situations: We removed the discomfort predictor from the proposed method, meaning that we only used the effectiveness predictor, which takes the situation and phrases as its input but without the discomfort input.

• Phrases-only: We removed the situation input as well as the discomfort predictor from the proposed method, meaning that we only used the phrases input for the effectiveness predictor.

• Baseline: Returns the average effectiveness value of the training data because this minimizes the MSE for the training data.

For all the above methods, we performed 25-fold cross-validation. That is, we split the dataset collected in Section 3 into 25 sets of 500 phrases each; thus, for each fold, there are 11,960 data used for training, 520 data for validation, and another 520 for testing. For the proposed method, for each fold of evaluation, we first trained the discomfort predictor and the effectiveness predictor using the same training data and validation data, and then tested the effective predictor with the test data. During the training, we chose the optimal model based on the best performance on the validation data according to the evaluation metric. We conducted an early-stop of the training when there was no improvement in the evaluation metric over the last five epochs of model updates.

### 5.2 Result

Table 4 shows the mean and standard deviation of the squared differences, averaged from all instances over all cross-validation trials, for a total of 13,000 instances. The mean squared differences resulted in 2.085 with the Baseline, 1.899 with Phrases-only, 1.846 with Phrases + Situations, and 1.805 with the Proposed Method (with Phrases + Situations + Discomfort).

**TABLE 4:** The results of mean and standard deviation.

Methods	Mean	SD
Baseline	2.085	2.305
Phrases-only	1.899	2.378
Phrases + Situations	1.846	2.391
Phrases + Situations + Discomfort	1.805	2.314

Significant differences were observed among all the methods. Normality was not confirmed based on the Kolmogorov-Smirnov test, so we applied the Wilcoxon signed-rank test. The difference between baseline and phrases-only was significant at p<.001 , and the effect size is 0.114. The difference between phrases-only and phrases + situations was significant at p=.006 , and the effect size is 0.0241. The difference between phrases + situations and phrases + situations + discomfort was significant at p<.001 , and the effect size is 0.0480.

These results indicate that including the features of phrases into the learning improved the accuracy of humor effectiveness predictions, and that including situation factor into the learning alongside phrases further improved this accuracy. Furthermore, the results show that predicting discomfort and utilizing it in the prediction process, along with phrases and situations, can further increase the prediction accuracy.

### 5.3 Case study

We used examples to investigate how the situation and discomfort contributed to better prediction of the effectiveness of humorous phrases.

#### 5.3.1 Contribution of situation

**FIGURE 5:**
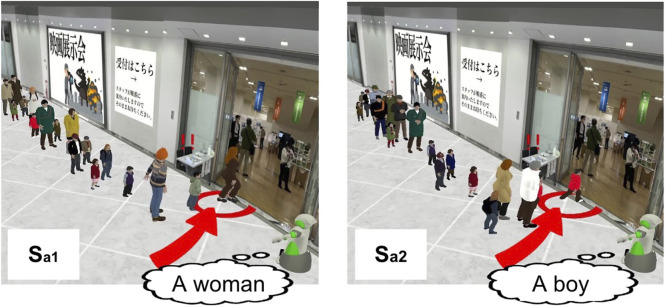
Two predicted situations for “The little ones are watching too, so please set a good example!”.

We evaluated the phrase, “The little ones are watching too, so please set a good example!” under two situations, $S_{a1},S_{a2}$ , shown in Figure 5. The proposed method predicted this phrase to have a higher effectiveness score, $E_{a1}^{\prime }=5.573$ in $S_{a1}$ , where an adult woman cut in the line. It predicted a rather lower score, $E_{a2}^{\prime }=4.073$ in $S_{a2}$ , where a young boy alone cut in the line. In fact, the ground truth for this adult case was $E_{a1}=6.000$ and the child case was $E_{a2}=4.000$ .

In contrast, the Phrases-only method, which did not use the situations for learning, predicted the effectiveness of this phrase for both situations to be $E_{a1}^{\prime }=E_{a2}^{\prime }=4.415$ , resulting in larger error than the proposed method.

#### 5.3.2 Contribution of discomfort

The phrase “Sorry, these customers are not sorted by how soon they will die” (as also shown in Table 3) would provoke large discomfort as its humor is rather offensive even if the situation would match with the phrase when the line cutting was conducted by an adult.

For this, the ground truth discomfort score was D=6.333 and the effectiveness was E=1.333 . The effectiveness predictor in the Phrases + Situations condition predicted its effectiveness at $E^{\prime }=4.872$ and the proposed method (Phrases + Situations + Discomfort condition) predicted the discomfort at $D^{\prime }=4.153$ and the effectiveness at $E^{\prime }=4.187$ . The error by the proposed method is smaller than the Phrases + Situations condition because the predicted discomfort values are higher than the mean value of 3.321 in the ground truth, decreasing the predicted effectiveness value.

## 6 The humor selection system using the developed predictors

**FIGURE 6:**
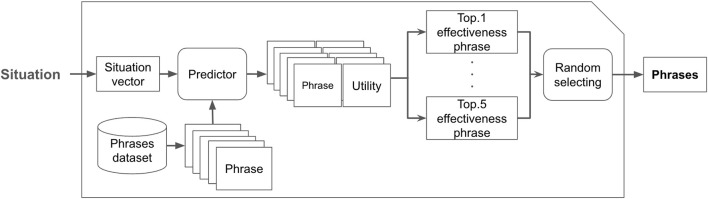
Overview of the humor selection system for line cutting.

We created a system that dynamically selects the optimal humorous phrase based on the situation when line cutting occurs using the humor effectiveness predictor proposed in Section 4. An overview of the system is shown in Figure 6.

In this system, when line cutting occurs, the situation is inputted. The inputted situation is converted into a situation vector using the process described in Section 4; information about the people in line and those line cutting, such as their gender, age, and number, is transformed into a vector representation.

Next, for each combination of this situation vector and the humorous phrases in the dataset in Section 3, we use the effect predictor proposed in Section 4 to predict the effect value for each phrase. This allows for the prediction of the effectiveness of all phrases in the dataset for the given line-cutting situation.

Finally, the obtained effectiveness values are sorted in descending order and the top five phrases with the highest predicted effectiveness for the given situation are randomly selected and outputted. It is desirable for the robot to have some variation rather than repeating the same phrase in similar situations. Therefore, we adopted a method of selecting a certain number of highly effective humorous phrases and randomly selecting from them. We selected the top five candidates because there is no significant difference in the predicted effectiveness values among the top five phrases for any situation and we expect that there will be no significant difference in effectiveness regardless of which one is chosen.

As an example, let us consider a situation where there is a line at an exhibition and a pair of an adult man and boy cut in. Here, we calculate the predicted values for all phrases in the dataset and show some of the sorted results in Table 5. For instance, the phrase “The little children are also lining up with excitement!” has a high predicted effect of 5.22. Moreover, as shown in Table 5, the top five phrases have predicted effectiveness values ranging from a maximum of 5.26 to a minimum of 5.11, indicating a small difference in effectiveness, and all are considered to have sufficiently high effectiveness. In contrast, a phrase like “I’m useless” has a low predicted effectiveness of 2.71 and would not be included as a candidate phrase.

**TABLE 5:** Examples of evaluating phrases using the humor effectiveness predictor.

Situation
* A line forms at the entrance of the exhibition
* The first group consists of an adult woman and a little girl
* The second group consists of an older man
⋮
* A pair of an adult man and boy cut in line

Humorous phrases	Effectiveness
Compared to the time you’ll spend viewing the exhibits the waiting time is trivial. So please wait a little longer and enjoy your viewing time to the fullest	5.26
The little children are also lining up with excitement!	5.22
The little ones are watching too, so please set a good example!	5.16
I’m sorry. In this exhibition, we have a system where you wait in line over there and enter in order	5.12
The small children at the back are also feeling confused so please line up at the end	5.11
⋮	⋮
The exhibition is not over yet.	3.72
⋮	⋮
I’m useless	2.71

## 7 User study

We described in Section 6 a method for selecting the optimal humorous phrases using a humor effectiveness predictor for line-cutting situations faced by robots. In this section, we verify through video experiments how effectively the humorous phrases selected by this humor selection system discourage the customers from cutting in line compared to typical non-humorous phrases. Participants in the experiment watch a video reenactment of line-cutting behavior and evaluate the effectiveness of the phrases under each situation.

### 7.1 Conducting a video-based study

While it would be ideal to evaluate the effectiveness of the humorous phrases selected by this humor selection system in actual situations, conducting such experiments is not feasible. If line cutting occurs, we should stop it as soon as possible. Additionally, it is reasonable to assume that people will refrain from cutting in line if they know they are being observed and recorded. Moreover, ensuring a sufficient number of trials to validate hypotheses would be extremely challenging.

Therefore, we opt for an alternative approach by conducting video experiments to simulate real-life scenarios. First, we filmed examples where customers cut in line, ignoring the robot’s instructions, to create reenactments in various situations. Second, we showed the participants these videos where the line-cutting customers were the same age and gender as the participants to put themselves in the shoes of the line-cutting customers. Third, the participants evaluated the effectiveness of the phrases of each condition. To ensure enough data by presenting phrases to a diverse range of individuals, we conducted the experiment using crowdsourcing.

In these videos, all customers standing in line and those cutting in line were portrayed by actors. This approach was chosen because it would have been difficult to collect a sufficient variety of real-life video data of line-cutting incidents due to time constraints and the need to obtain permissions for video usage. Additionally, ideal data was required for this video experiment, ensuring that evaluators could easily recognize the low moral behavior just by watching. The use of these video data without any alterations to faces or voices has been fully consented to by all actors, under the condition that they are only used within the scope of this research.

### 7.2 Hypothesis and prediction

It is not yet clear how effective a security guard robot will be at discouraging low moral customers from cutting in line by using the humorous phrases selected by our proposed method. However, such a possibility is strongly suggested by previous research. We use humor in many daily-life situations to persuade or convince others. The effectiveness of humor is evident even in fear appeals, where defensive reactions tend to increase (Mukherjee and Dubé, 2012). Furthermore, there is evidence suggesting that robots can achieve similar effects, such as audience happiness and favorable impression of robots, by using humor (Katevas et al., 2015; Hayashi et al., 2008).

Based on these prior works, we hypothesized that, when our proposed system works correctly, the robot will select effective humorous phrases, use them to discourage line-cutting behaviors, and achieve better results compared to typical non-humorous phrases. Therefore, our hypothesis for this experiment is as follows:

• Prediction 1: People who hear (see in speech text bubble) the humorous phrases selected by the proposed method are more discouraged from continuing cutting in line compared to typical non-humorous phrases.

### 7.3 Method

#### 7.3.1 Condition

We compared the following two conditions. For each condition, a robot in a video utters a phrase with AI voice to discourage participants from continuing line cutting.

• Typical phrases The robot uses a typical non-humorous phrase that normal security guards tend to use in actual situations. Such phrases are selected from a dataset of typical non-humorous phrases collected in Section 3. Unlike the dataset of humorous phrases, multiple writers frequently used the same phrases for multiple situations in the non-humorous dataset. Therefore, we selected the frequently used phrases as typical phrases; we sorted the frequency of non-humorous phrases and selected the top five phrases for this experiment.

• Humorous phrases The robot uses humorous phrases suitable for the line-cutting situation of each reenactment video. To select optimal humorous phrases, we used the humor selection system proposed in Section 6.

Furthermore, we ensured in advance that the selected phrases were not discriminatory or contrary to moral principles. If the robot were to use phrases that pose ethical problems during the experiment, it would not only cause significant psychological distress to the participants but also severely damage trust in both the robot and its researchers. Therefore, we must carefully consider preventing the robot from using such phrases. Guided by these principles, we aimed to develop a system where the robots do not use such phrases through discomfort prediction and learning. Indeed, no such phrases were selected in this experiment.

#### 7.3.2 Participants

We gathered 351 online adult participants through the same crowdsourcing company described in Section 3. All participants were compensated 300 JPY. The data collected are anonymized and do not identify individuals, and all rights to the data belong to us.

Out of these participants, only one failed to provide correct answers and was thus excluded. This very low rejection rate is believed to be due to the company’s practice of sourcing qualified workers using feedback from past jobs, which tends to attract individuals more committed to their work compared to those recruited from other platforms. Additionally, the fact that this video experiment is relatively simple and short may have contributed to the low rejection rate. Therefore, we analyzed the responses of the remaining 350 participants.

Furthermore, participants were asked to provide information regarding their gender (man/woman) and age (20–49 years old/60 years old and above), and whether they usually go to an exhibition with another person. If they usually go with another person, they were also asked to provide information regarding the companion’s gender (man/woman) and age (elementary school-age/20–49 years old/60 years old and above). The breakdown was as follows: 168 men and 182 women, 275 participants aged 20-49, and 75 participants aged 60 and above. This distribution of participants follows that of the crowdsourcing platform. We excluded children’s participation from this experiment because it is difficult to adequately ensure the validity of children’s responses.

#### 7.3.3 Video stimuli

The experiment participants watched three types of videos. In the first video, a customer who is the same age and gender of the participant cuts in line and the participant is required to think as the customer. In the second and third videos, the robot persuades the participants not to cut in line. These videos are presented under the two conditions of typical phrases and humorous phrases, with the order randomized for each participant.

**FIGURE 7:**
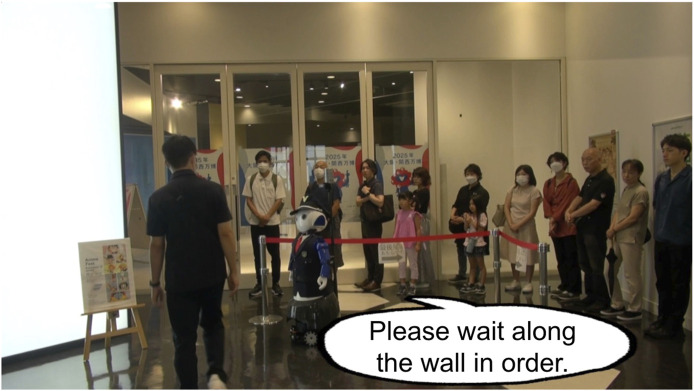
Example of low moral behavior video where a man ignores a guard robot and cuts in line.

The first video depicts an exhibition where people are waiting in line at the entrance. A security guard robot stands at the front of the line, guiding people to the end of the line. Then, a customer or a pair of customers appears and attempts to cut in line. Despite the robot’s instruction to “Please wait along the wall in order” (Figure 7), they ignore the robot and try to cut to the front of the line.

Before watching the videos, the experiment participants answered questions regarding their own attributes, such as their age, gender, and information about their companion. Based on these responses, we displayed the video that the participant is most likely to role-play. A total of 22 different scenarios were prepared, covering all possible combinations of responses.

**FIGURE 8:**
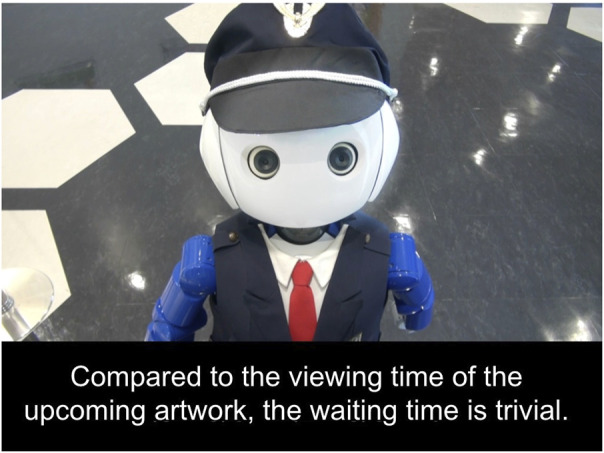
Example of guard robot video.

In the second and third videos, the participant is shown the security guard robot persuading from a first-person perspective. This includes the robot’s movements, voice, and text displaying the phrases. The phrases were selected according to the two conditions explained in Section 7.3.1: typical non-humorous phrases and humorous phrases. Figure 8 shows an example.

#### 7.3.4 Measurement

For each phrase, we measured the likeliness of discouraging the participants from continuing line cutting. The participants were asked to rate the phrase on a 7-point Likert scale with the question:

• Effectiveness: How likely would you be discouraged from continuing line cutting?

A rating of 1 indicates “absolutely would not be discouraged” and 7 is “absolutely would be discouraged.”

#### 7.3.5 Procedure

**FIGURE 9:**
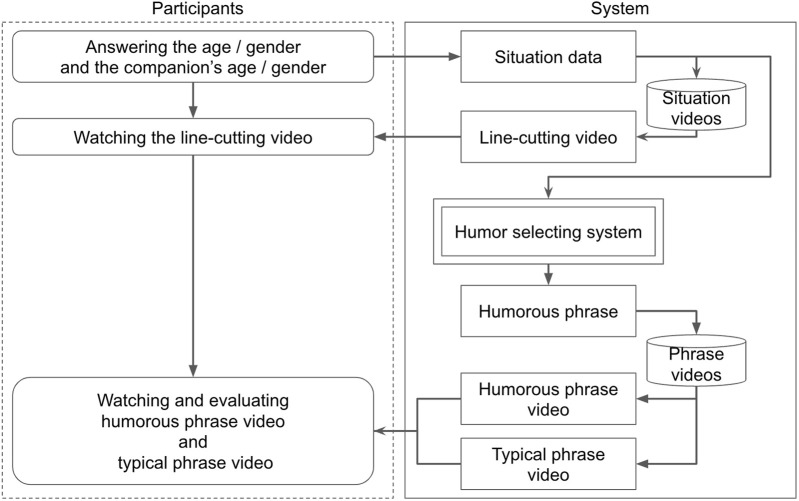
Overview of the video experiment.

The overall procedure is illustrated in Figure 9. The video experiment followed three steps: In Step 1, the participants answered questions about their gender, age, and whether they usually attend exhibitions alone or with someone else. In Step 2, they watched a video showing a scenario of a customer or a pair of customers cutting in line. In Step 3, they watched two videos of the robot persuading customers not to cut in line and evaluated the phrases for each of the two conditions.

Step 1: Participants in the experiment answer questions regarding their own attributes, such as their age, gender, and their companion’s information. On the system side, using the attributes provided by the participants (and those of their companions), the system selects a video from 22 reenactments of line-cutting behavior that best matches the participants’ responses. The selected video is presented to the participants.

Step 2: Participants watch the reenactments of line-cutting behavior and are asked to respond to why they would cut in line in the situation shown in the video. We present this question assuming that participants can engage in deeper role-play by answering it. Additionally, we present a question to verify the accuracy of the participants’ responses, asking them to answer the phrase spoken by the robot in the video. They select from four choices. The correct answer is “Please line up along the wall.”

Step 3: Participants watch videos of the robot speaking phrases under two conditions: typical non-humorous phrases and humorous phrases selected by the humor selection system. Humorous phrases are selected based on the attributes as input and output by the humor selection system proposed in Section 6. Typical non-humorous phrases are randomly selected from the top five phrases with the most responses in the dataset of typical non-humorous phrases collected in Section 3. Within the system, the videos in which the robot speaks these phrases are selected and presented to the participants. The participants first watch a video on one of the two conditions and evaluate the phrase. Additionally, they are asked to answer the phrase spoken by the robot in the video, similar to in the previous question. After answering these, they watch the video for the other condition and respond to the same questions.

The experiment was done as a within-subject study. The order of watching the videos for each condition is counterbalanced to account for order effects. That is, half of the participants watch the video of typical non-humorous phrases first, while the other half watch the video of humorous phrases selected by the humor selection system first.

### 7.4 Result

**FIGURE 10:**
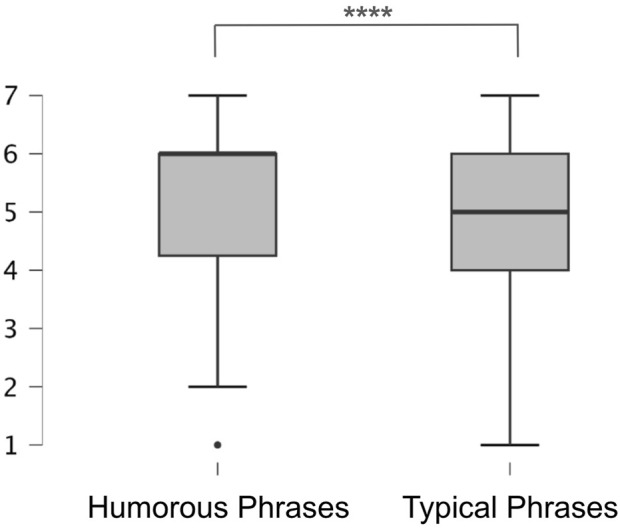
A box plot showing the distribution of the effectiveness in the two conditions: the Humorous Phrases (Q1 = 4.25 and Q2 = Q3 = 6.00) and the Typical Phrases (Q1 = 4.00, Q2 = 5.00, and Q3 = 6.00)

Figure 10 shows the effectiveness results. We applied the Shapiro-Wilk test, and its results show that the distribution deviated significantly from normality. Additionally, the data for these two conditions consist of paired evaluation values assessed by the same participants, making these data correlated.

Therefore, we applied the Wilcoxon signed-rank test, which revealed a significant difference ( z=6,254 , p<.001 ) between typical phrases ( μ=4.65 , σ=1.52 ) and humorous phrases ( μ=5.27 , σ=1.47 ). Therefore, prediction 1 was confirmed: people who hear the humorous phrases selected by the proposed method are more discouraged from continuing cutting in line compared to typical non-humorous phrases.

## 8 Discussion

### 8.1 Implications

We discuss the utility of effectively employing humor (8.1.1) and discomfort (8.1.2) in robot tasks, as demonstrated in this study. We also discuss the usefulness of using crowdsourcing as a research method (8.1.3). Furthermore, address the necessity of considering cultural context, as suggested in this study (8.1.4).

#### 8.1.1 Humor

Although it is currently only a video-based evaluation, our study suggests the possibility to use humor in persuading people in situations where typical phrasing does not work well. This potential effectiveness extends not only to security robots but also to various other HRIs involving persuasive-like tasks. For example, robots that act as facilitators in human discussions could use humor to defuse tense situations. Moreover, when using robots as teachers or exercise instructors, humor might be employed to motivate learners.

#### 8.1.2 Discomfort

Our study demonstrated the importance of considering human discomfort while computing the predicted effectiveness. This idea - to consider potential discomfort - could be applicable to various tasks and, more broadly, in selecting robot utterances. Actually, some previous studies used the concept of discomfort to design polite or social behavior, and have revealed its positive effect. For example, polite robots were perceived as friendlier, fairer, and as acting in a more appropriate way (Inbar and Meyer, 2019). In another example, social-focused robots were perceived to have more natural and pleasant behaviors compared with service-focused robots, and perceived as having a more persuasive effect on the users’ choices (Rossi et al., 2022). Furthermore, learning discomfort makes it possible to design the robot in a way that it does not select phrases that pose ethical concerns.

On the other hand, there may be situations where intentionally increasing discomfort proves to be effective. In this study, we focused on behaviors that require compliance with a single sentence, which led to the implementation we used. However, the data obtained included examples that showed high discomfort but also high effectiveness (e.g., “The exhibit must be really fascinating if you’re cutting cut in line to see it”). This suggests that a strategy of using high discomfort to persuade, depending on the situation, might be necessary. In fact, previous study has shown that utterances which would cause discomfort when performed by humans have possibility to improve robot’s task performance when performed by robots. Naito et al. have shown examples where a robot using direct, typically rude behavior have better customer service outcomes than a robot using traditional indirect behavior (Naito et al., 2023). Additionally, employing a flexible approach to adjust discomfort in interactions requiring multiple sentences might also be an effective strategy.

#### 8.1.3 Crowdsourcing

We successfully collected a variety of humorous phrases, for which the use of crowdsourcing was substantial. Crowdsourcing enabled us to collect many creative humorous phrases, as well as impression data on the robot’s humorous phrases from diverse individuals. Previous HRI studies have also demonstrated the uses of crowdsourcing. Breazeal et al. used crowdsourcing as a rich source of interactions (Breazeal et al., 2013). Inamura et al. proposed crowdsourcing as an alternative method for the COVID-19 pandemic (Inamura et al., 2021). We believe our case adds on the previous success of using crowdsourcing and would imply further possibility for use in diverse scenes.

#### 8.1.4 Cultural context

Humor is deeply intertwined with cultural context. For instance, the phrase ‘If you’re not good, Santa won’t come’ is perceived differently depending on the culture. In Japan, where Christmas is accepted as an event with little religious significance, it may be taken as humor. However, in countries with a strong Christian faith, this phrase could be perceived as very harsh for children. Moreover, in cultures where there is no Christmas tradition, it would be completely meaningless.

Additionally, what constitutes humorous content or what speaking style or attitude is considered humorous may largely depend on cultural factors. A phrase recognized as humorous in one culture may not necessarily be perceived as humorous when translated into another language. Therefore, when using humor, it is essential to carefully consider the types of humor that are preferred in the specific cultural context and to ensure that what is being used is genuinely recognized as humorous within that culture.

### 8.2 Limitations and future works

Our effectiveness prediction framework was only tested with a limited target and online evaluation. Hence, careful consideration is needed before using it with a robot. As the limitations of this study and future works, we discuss particularly focusing on situation vectors (8.2.1), as well as other factors (8.2.2) and the potential for applicability in reality (8.2.3).

#### 8.2.1 Situational recognition, expression and relationship with humor

##### 8.2.1.1 Recognition

Firstly, the parameters of the situation must be carefully considered. In this study, we conducted experiments under the assumption of ideal situation recognition without using robot image recognition.

In fact, as an example of the latest image recognition technology, the system proposed by Kumar et al. successfully determines age with an accuracy of 83.26% and gender with an accuracy of 95.31% (Kumar et al., 2022). This system classifies age into very detailed categories (0–2, 3–7, 8–13, 14–20, 21–36, 37–60, 60+) based on facial photographs. In practical applications, it is possible to also consider additional information such as height and voice. For broader classifications like those used in this study (child/adult/aged), the system is believed to achieve high accuracy without compromising its performance.

However, in practice, it is necessary to account for the possibility of image recognition errors by the robot. In scenarios where misrecognition is frequent, it may be preferable to use more general, context-independent humor rather than context-specific humor. As an alternative, if recognition is ambiguous, predicting discomfort and effectiveness across multiple context patterns and avoiding those with undesirable discomfort or effects could be considered.

##### 8.2.1.2 Expression and machine learning

About the role of contextual information on humour effectiveness, it remains unclear whether these parameters are enough to represent the situation in the real world. There may also be other important parameters beyond those considered in this study. For example, while this research simulated scenarios with a maximum of two people, in reality, there could be situations where a large group of children cuts in line or multiple groups cut in line simultaneously. In the simulation, only specific form of line and exhibition was included. It may be better to ask the evaluator why certain phrases are humorous in specific situations, through creating dataset. This will make it possible to clarify which situational elements influence the perception and effectiveness of humor.

Furthermore, we should discuss that the effect size from learning situations in the proposed machine learning approach is smaller than expected. In this study, the training dataset included 460 types of phrases, while the validation and test datasets contained 20 types each. Even with 13,000 data, it is possible that the relationship between phrases and situations was not learned sufficiently. However, it has been confirmed that learning from situations significantly improves accuracy, particularly as demonstrated in Section 5.3, where evaluations of phrases tailored to specific situations have improved. Moreover, this improvement in phrase evaluation accuracy for different situations is also observed in the system described in Section 6, confirming the effectiveness of situation-specific phrase selection in the proposed system.

Additionally, while this study selected humor tailored to specific situations, it is not clear whether the chosen humor represents the best possible phrase for that situation or if it is within the top 5% of effective phrases. Due to the limitation of data, this study does not have data where all phrases have been evaluated for each target situation. We have combined phrases with 13,000 situations, and it is not feasible to obtain evaluation data for all combinations of these phrases and situations.

##### 8.2.1.3 Relationship between situation and humor

We also need to discuss whether humor that is more contextually specific has a greater effect in the future.

Firstly, we consider that generic humor which can used all situation of all low moral behavior does not exist, but it has been suggested that there may be humor that behaves generically in response to line-cutting at an exhibition. For example, the phrase “Don’t worry. The exhibition won’t run away. Please wait patiently at the back.” is possible to be “generic”, only for cutting in line at an exhibition. Therefore, it could be said that this phrase is context-dependent but generic within this situation. However, although we collected the limited data in this study, such phrases do not consistently show high effectiveness (for example, not all phrases receive a rating of 5 or above on a 7-point scale for every situation).

We consider that humor exists on a spectrum of contextual specificity, and that selecting humor with higher contextual specificity may lead to greater effectiveness. While we observed trends suggesting this from the collected data, we did not obtain sufficient data to demonstrate objective differences.

Therefore, demonstrating the effect of humor based on its level of contextual reference in the future will likely emphasize the importance of learning about contextual situations.

#### 8.2.2 Influence of other factors on humour

We need new data collection for humorous phrases to apply our framework for different low moral behaviors and different languages and cultures. Humorous phrases would be more effective in combination with a robot’s bodily motion. Additionally, while we collected phrases from writers this study, we believe that obtaining phrases from other professions, such as comedians, could enhance the quality of the phrase dataset.

We also need to consider the effect of robot’s appearance, such as cuteness. Previous research has shown that the appearance of robots, such as their animal-like or human-like features, can enhance their likability (Li et al., 2010). Additionally, it has been demonstrated that the cuteness of service robots increases consumers’ willingness to engage with them (Guo et al., 2024). The appearance of robots plays a significant role in the impact they have, and the element of cuteness may be an invisible factor that influences the effectiveness of humor. This issue is highly suggestive in the context of how social robots use humor to persuade others and needs to be clarified in future research.

#### 8.2.3 Applicability in reality

We only evaluated the effectiveness via online evaluation; thus, even if people in the real world would be similarly discouraged by the selected humorous phrases with our framework, further investigation is needed to determine whether they really stop low moral behavior.

Moreover, just as there are differences in persuasive performance between humans and robots, the effects and impressions of using humor in persuasion could also vary between them. Clarifying this could provide insights into the advantages and considerations of using humor with robots. Additionally, exploring not only verbal humor but also other methods such as sound, light and appearance could reveal new ways in which robots might succeed in persuasion in ways that humans cannot.

## 9 Conclusion

We proposed the use of humor by security guard robots to address line cutting during their duties. To overcome the constraints of real-world data collection, we utilized crowdsourcing and built a large-scale dataset using simulators. With this dataset, it became possible to predict the effectiveness of humor using machine learning. Additionally, we discovered the importance of considering the situation and discomfort in predicting effectiveness. Furthermore, using this predictor, we built the system to select the optimal humor from the dataset according to the line-cutting situation. Through video experiments, we demonstrated that people who hear (see in the speech bubble) the humorous persuasion selected by the proposed method are more discouraged from continuing line cutting compared to typical non-humorous persuasion.

## Data Availability

The raw data supporting the conclusions of this article will be made available by the authors, without undue reservation.
